# What matters most to the patient – a qualitative study of older patients in a geriatric ward

**DOI:** 10.1186/s12877-026-07555-y

**Published:** 2026-04-30

**Authors:** Åsa G Andersson, Kim Jackwert, Karin Hugelius, Lisa Kurland

**Affiliations:** 1 Department of Geriatrics, School of Medical Sciences, Faculty of Medicine and Health, Örebro University, Örebro, SE70182 Sweden; 2 Center of Medical Education, Falu Lasarett, Region Dalarna County, Falun, SE79182 Sweden; 3 Faculty of Medicine and Health, Örebro University, Örebro, SE70182 Sweden; 4 School of Medicine, Örebro University, Örebro, SE70182 Sweden

**Keywords:** What matters most, Patient-centred, Frailty, Older

## Abstract

**Introduction:**

The ageing of the population is expected to increase the costs and consumption of health care. Care should be patient-centred. Patient-centred care (PCC) has been shown to increase the quality of life for older patients living with frailty, as well as reduce health care costs. As one of the core principles of PCC, it is important to investigate and consider patients’ wishes regarding their care.

**Aim:**

The aim of this study was to investigate and identify factors and information that older patients find important when planning for their care.

**Methods:**

Sixteen patients at the geriatric ward at Örebro University Hospital in Sweden were interviewed using focus group discussions. The patients were ≥ 65 years of age, spoke Swedish, and were cognitively and physically able to participate in a focus group discussion. The interviews were recorded, transcribed, and analysed using content analysis.

**Results:**

Three main categories were formed to summarize what mattered most to the patients when planning their care were that they maintained autonomy, that no harm was done and that the care had a holistic approach. These were further divided into the subcategories: the right to be well informed, taking part in planning of their care, having accessible care and a feeling of safety and security, considering emotional wellbeing, including relatives and close friends and maintaining their wellbeing.

**Conclusions:**

What mattered most to the patients was closely related to the World Health Organization’s recommendations for ethical and good quality health care. Generally, the patients agreed on what mattered most. Further studies are needed to enrich the understanding of what is important to older patients.

**Supplementary Information:**

The online version contains supplementary material available at 10.1186/s12877-026-07555-y.

## Introduction

Older people, often defined as people aged 65 and older, constitute approximately 20% of the population in developed countries. This proportion is expected to increase as the world is experiencing global population ageing due to increasing life expectancy [1, 2]. Ageing of the population is expected to increase the cost and consumption of health care [3–5], especially through increased need for long-term care including assisted living [4, 6]. This increase in the need for health care has been found to be due largely to the geriatric conditions of multimorbidity and frailty, which are both more common with increased age [7]. Frailty is thought to occur because of a lack of adaptive and resistive capacity, along with reduced biological reserves of the body [8–10].

To meet the needs of the ageing population, a health care system needs to be able to provide patient-centred care (PCC) [4, 11]. This aims to form a holistic picture of the patient and include them as an active participant in their own care [12]. A holistic approach is favourable for people with multimorbidity as they are at risk of fragmentation of care, since their care in the current health care system is typically split between several caregivers and institutions [13]. Implementing PCC is also beneficial for attending to the multiple aspects of frailty, which compromise daily life and independence [11]. The effect of PCC can be measured using patient-centred outcomes. Patient-centred care has been shown to lead to a reduction in health care costs, improvement in symptom burden, and positive health outcomes [12, 14].

It aims to put less emphasis on illness, and more focus on the person and their needs and wishes [12]. This is achieved by designing a care plan based on what matters most to the person, instead of focusing on clinical guidelines. It is therefore of interest to explore the patient’s wishes for their care.

There is, however, a paucity of knowledge concerning what matters most to older patients in the health care system, in particular as expressed with their own words. Previous studies regarding what matters most to patients have focused on either a more generalized population, rather than studying older patients living with frailty specifically, or focused on a specific disease or event, or have not included the patients themselves in the study [14]. Several studies have focused on asking older patients regarding emergency care [15–18]. To the best of our knowledge, only two previous studies have included the patients themselves when investigating the requests of older patients in a general setting [19, 20]. However, the first study only included homebound persons [19], and the second provided seven goals for the participants to rank, rather than asking them freely what they thought mattered most [20]. It is therefore important to study what older patients request and wish for in relation to their care, using their own words.

To better prepare for implementation of PCC in order to improve care for older patients, it is of importance to investigate what matters most to them regarding their care in general. Additionally, by Swedish law, a patient has the right to participate in decisions regarding their own care [21], which further underlines the relevance of this study. The benefits of PCC, demonstrated by both patient-centred outcome measures and economic factors [11, 13, 14, 22], make this a very topical research subject in view of the ageing global population and the resulting financial strain on health care.

The aim of this study was to investigate and identify factors and information that older patients find important when planning for their care.

## Methods

### Study design

An inductive qualitative study was conducted.

### Study participants

Patients admitted to a geriatric ward in Örebro University Hospital between September and October 2024 were eligible. Patients were included consecutively and if they were ≥ 65 years old, spoke Swedish fluently, and had provided written informed consent. The following exclusion criteria were applied: age < 65 years, prior cognitive impairment documented in medical records, patients who due to illness could not participate in focus group discussions (FGD) and inability to understand and express themselves in Swedish without interpretation. The patients received verbal and written information. Informed consent was obtained, and written consent was required to participate.

### Sample and collection of data

Focus group discussions (FGDs) were used to collect data [23]. Five FGDs were carried out with two to four patients in each group, in total including 16 patients. Studies have shown that focus groups add value when interviewing on emotionally charged topics, since the group may serve as social support for the study participants [24].

The number of participants in each FGD varied based on the number of eligible patients willing to participate among the patients treated in the geriatric ward. The concept of information power [25] was used to determine the study sample size. Given the aim, the inclusions- and exclusion criteria, a total sample size around 15 participants. A broad study aim and no use of established theory suggested a higher number of participants, while a dense sample specificity and strong quality of dialogue suggested a smaller number needed. The analysis strategy, being cross-case, suggests a larger number, but also being exploratory eventually suggests a smaller number [25]. All interviews were conducted in Swedish and took place in the geriatric ward between the 3rd of September and the 25th of October 2024. The first author (ÅA) acted as moderator, leading the discussions. The second author (KJ) acted as co-moderator, observing the discussions, and operated the recording equipment. Two interviews were carried out by ÅA alone. A semi-structured interview guide was used during the FGDs, which started with an opening question: “What matters most to you when we plan for your care?” The guide also contained several open-ended questions based on this principal question, as well as a few additional questions for further exploring a topic. See Additional file 1. The guide was tested in the pilot FGD and as no changes were made, the pilot FGD was also included in the results. The median duration of the FGDs was 35 (range 16–53) minutes. The recordings were transcribed manually by the second author (KJ) using naturalized transcription where non-verbal cues were not included [26].

Patient characteristics are presented in Table 1. A brief review of their medical records was conducted using a standardized template. The primary diagnoses using ICD-10 diagnostic codes were sorted into three categories according to prevalence: internal medicine, orthopaedic and surgical. Comorbidity was evaluated by means of the Charlson Comorbidity Index (CCI), a weighted index estimating 1-year mortality based on comorbidities. The CCI consists of a numeric scale based on the presence of specific diagnoses as well as the patient’s age [27]. Depending on the score, the patients can be categorized as mildly ill (CCI score 1–2), moderately ill (CCI score 3–4), or severely ill (CCI score ≥5) [28]. An online CCI calculator [29] was used by the examiners to determine the CCI, following guidelines outlined in an article made specifically for Swedish registers [30]. Frailty was assessed according to a verified Swedish translation of the Clinical Frailty Scale (CFS) version 2.0 [31]. This is an ordinal scale ranging from 1 (very fit) to 9 (terminally ill). The CFS is commonly used in clinical practice to assess frailty, and it is easy to administer and has shown good predictive ability and high inter-rater reliability [9, 32, 33]. The patients’ habitual level of frailty was assessed 14 days prior to admission to hospital in accordance with recommendations [9, 34]. These CFS-scores were based on information from the medical records [35].

**Table 1:** Patient characteristics

Age, *n* (%)	
65–79 years	5 (31)
80–89 years	8 (50)
90–99 years	3 (19)
Gender: female, *n (%)*	9 (56)
Ordinary housing, *n (%)*	16 (100)
Lives alone	9 (56)
Main diagnosis (ICD-10), *n (%)*	
Internal medicine	8 (50)
Orthopaedic	5 (31)
Surgical	3 (19)
Secondary diagnoses, md (range)	6 (2–14)
Comorbidity^1^, *n (%)*	
Mildly ill	0 (0)
Moderately ill	8 (50)
Severely ill	8 (50)
Clinical Frailty Scale^2^, *n (%)*	
1–3	7 (44)
4	3 (19)
5	1 (6)
6	3 (19)
7	1 (6)
8	1 (6)

^1^According to the Charlson Comorbidity Index (CCI)

^2^Clinical Frailty Scale taking in account the 14-day rule applied by Rockwood et al. to describe habitual status

*n*=16

### Data analysis

The transcripts of the FGDs were analysed using inductive content analysis [23]. After participating in most of the FGDs, and transcribing and listening to all recordings, the second author (KJ) read through the transcripts to become immersed in the text before starting the analysis. Meaning units were identified and extracted, condensed, and coded in separate code sheet. Transcripts were revised several times to ensure that no relevant information was being left out. The first author (ÅA) reviewed the transcripts for relevant meaning units and, together with KH, reviewed the code sheets and discussed the codes with KJ.

**Fig. 1:**
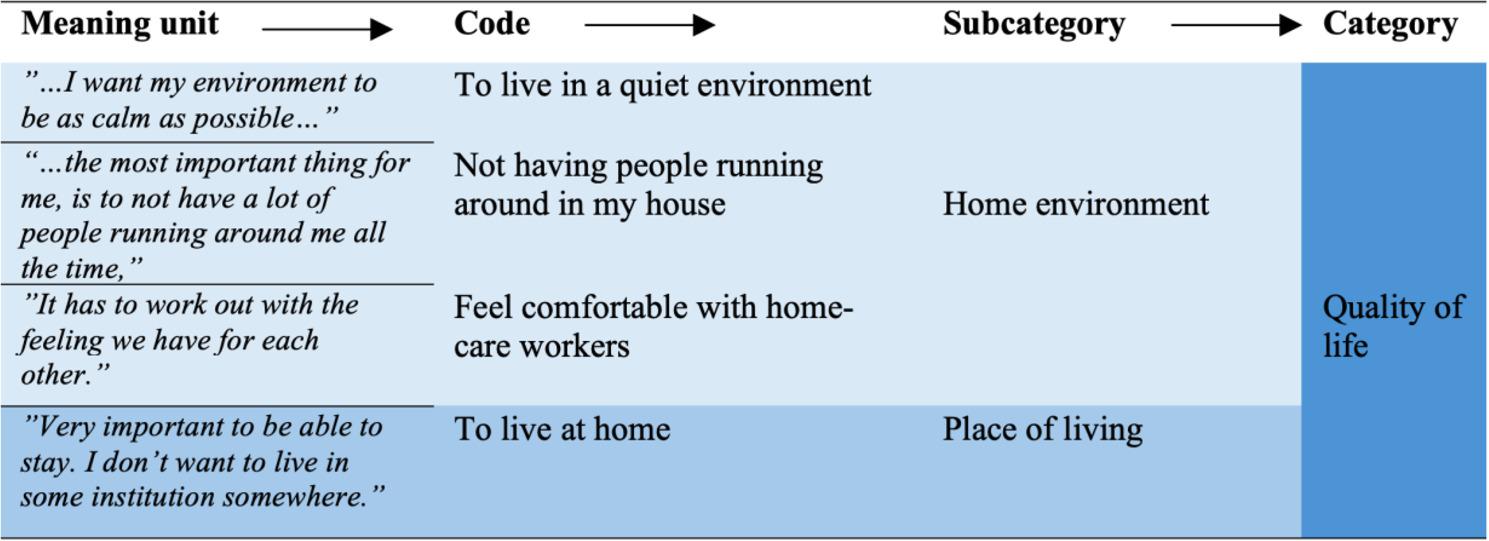
Extract of analysis progress

Thereafter, the 119 codes were organized into 29 preliminary subcategories based on their manifest content. This process was continued until all codes were sorted into subcategories that were homogeneous and heterogeneous, e.g. when no codes appeared in several subcategories and no codes were not included in any subcategory. These subcategories were thereafter organized into eight subcategories describing their latent content (as examples of the process in Fig. 1), and in turn formed three main categories (as outlined below). Categorization was conducted in English by KJ. The process and abstraction was revised and discussed by the entire team to ensure validation.

## Results

In total, 22 patients were invited to participate in this study. Out of these, 16 agreed and gave verbal and written consent. There was no statistically significant difference in age between those who gave consent and those who did not. The median age was 81 years in both groups, *p* = 0.59. The difference in sex between those who gave consent and those who did not was likewise not statistically significant, *p* = 0.16.

Patient characteristics are presented in Table 1. The median number of days between admission to the geriatric ward and the FGDs was 7 (range 1–23) days. Prior to admission to the geriatric ward, four patients had home care, while eleven had been living unassisted. For one patient, no information about home care could be found in the medical records. Two patients had received information about palliative care. Treatment restrictions in Do-not-resuscitate or attempt cardiopulmonary resuscitation forms were set for nine patients, and intensive care restrictions for five patients.

**Fig. 2:**
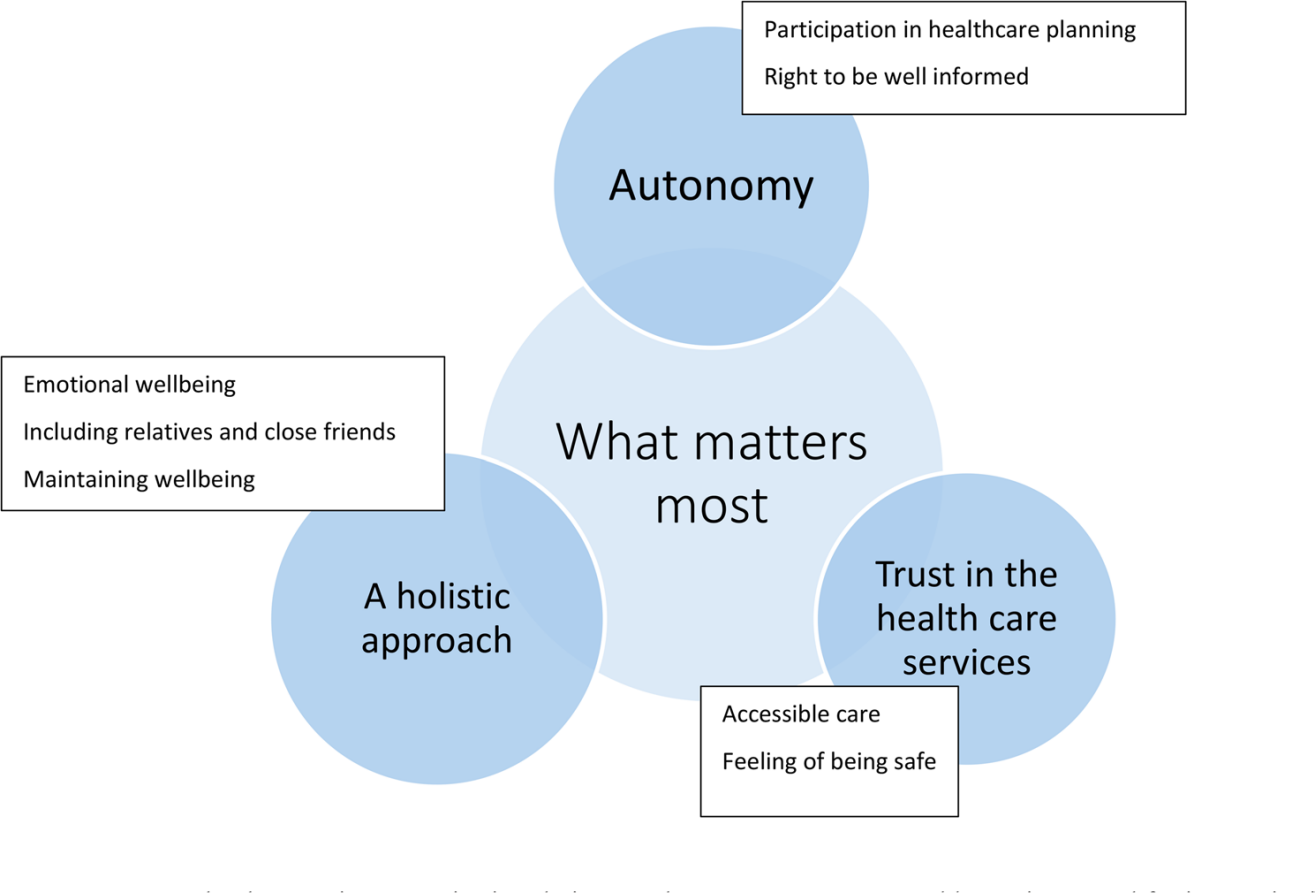
The three main categories in relation to what matters most to an older patient cared for in a geriatric ward. Also presented are the eight subcategories

Based on the FGDs, three main categories were formed to summarize what mattered most to the patients when planning their care: autonomy, do no harm and a holistic approach. The subcategories to “Autonomy” were to be well-informed and participation in healthcare. The subcategories to “Do no harm” were accessibility, safety and security. Lastly, the subcategories to “A holistic approach” were emotional health, inclusion of relatives and close friends. The main categories and their subcategories are presented in Fig. 2 and in the following text.

### Autonomy

#### Participation in healthcare planning

The patients expressed the wish to take part in the planning of their care as much as possible, and especially that decisions would be made in consultation with them. During this time, the patients expressed the wish to both physically attend and be actively included in the discussions. This wish applied to both in- and outpatient care. The patients also wished for all active parties to participate in the discussions, instead of having many small discussions with many different parties.

*I have no knowledge [of the medical field]*,* but I would want to participate and then I would be able to ask the question myself*,* “Why are we doing that?”* FGD 3.

*[…] I want to participate in my care as long as I*,* as my intellect is intact. And [as long as I] understand.* FGD 1.

Often, patients described that important points had been left out when making decisions, leading to unnecessary struggles for them. Often this related to events involved in transfer between departments or from inpatient to outpatient care. They wished to be able to communicate their wishes and needs first-hand to all caregivers involved.

#### The right to be well-informed

The patients wished to be adequately, continuously and transparently informed of their care and future interventions. They argued that this was important to make participation possible, as participating is difficult when information is being withheld. Being well-informed also gave a sense of security and trust.

*In my experience*,* what matters most to me is to be informed about what [the health care professionals] are doing and how*,* what they are planning to do*,* and that they communicate with me in words that I understand. And that they do not talk over my head*,* but with me.* FGD 1.

*[I want them] to explain why I am moved [from the ward]. Not just “Hey ho*,* let’s go!* FGD 1.

### Trust in the health care services

#### Accessible care

The patients also described the importance of accessible care for all and access to a contact person. They expressed a wish for access to treatments and diagnostic methods in time, and many were frustrated at the effort it took to be taken seriously and to finally get the right treatment.

*If you’re ill*,* you should be very ill. Because then [the health care system] can accommodate you.* FGD 1.

*[…] I need to have a phone number. If things don’t work out*,* or if things work out well – or if there are any changes*,* contact that [number]. So that I don’t sit there at home*,* like*,* ”Who should I call?”* FGD 1.

The subject of available hospital beds was discussed in several focus groups.

*[…] But they needed my bed. And I hate that saying.* FGD 1.

#### Feeling of being safe

The patients wanted to feel safe and secure. The importance of being able to trust the caregivers was emphasized several times. Some patients had experience of receiving inadequate care and had suffered as a result; others just expressed their wish to become better again by whatever means the health care professionals judged to be necessary. Another important matter was safe transitions, for instance between departments or between in- and outpatient care. Many times, the patients felt that their needs could not be communicated, as mentioned above, leading to unnecessary suffering.

*Yes*,* like for one instance when they discharged me on a Friday*,* [saying*,* ] “You may call home care on Monday.” There I was*,* with a broken leg. […] I had no food at home*,* I had nothing.* FGD 1.

The patients also expressed their wish to choose their place of death. Most wanted to die at home, where they felt safe, surrounded by family. Time to adjust to new information, an opportunity for a follow-up visit, and continuity were other factors they mentioned, all contributing to a feeling of security.

### A holistic approach

#### Emotional wellbeing

The patients also wished for health care workers to consider their emotional wellbeing, even when their primary complaints were physical, and not to treat patients differently because of stereotypes.

*Even if I am a bearded man in my seventies*,* I still have feelings.* FGD 1.

#### Including relatives and close friends

Including of relatives and close friends also mattered to the patients. They expressed their wish to include their relatives and friends in decisions and discussions about their care, both as this gave them a feeling of security, and because they felt that their relatives were part of their care. The patients also pointed out that they were sometimes too ill to properly inform their relatives themselves, so they wanted the health care workers to do this.

*Yes*,* I want a relative to be present*,* so they do not have to ask later.* FGD 3

*[…] I don’t want to see that only health care professionals make decisions about my life or upcoming situations; I want [my closest relatives] to be included in the entire process and to actively take part.* FGD 5.

The patients also wanted their relatives to be supported in dealing with the illness of their loved one, both emotionally and practically, with sufficient adjustments for life to work out at home.

#### Maintaining wellbeing

The patients expressed the need for a holistic approach when planning for their care, and the desire to maintain their wellbeing was a central topic in the FGDs. They asserted the importance of good quality sleep, pain management, and independence, as well as the importance of living conditions. To be able to live at home as long as possible was expressed as a recurring wish in the FGDs and any adjustments that made this possible were important to the patients. The patients pointed out that these adjustments had to be made at the right time for them to be useful.

*But the adjustments need to be done before I get home*,* you know. I might not even be able to get through my front door.* FGD 1.

Apart from adjustments in their daily lives, aiming to improve their ability to be independent, other adjustments for improved wellbeing during hospital stays were mentioned, such as the need for adjusting the means of communication when taking care of a patient with impaired hearing.

The patients also expressed the importance of attentive care, with helpful carers that listen to and respond to their needs and wishes. In all FGDs the wish to be taken care of while in hospital was mentioned. Patients wanted to be seen, heard and taken seriously. They wished for health care workers to take their time when seeing them, and to act professionally. Feeling welcomed and understood, and being treated well matters to patients.

*[…] that they take good care of me in the ambulance. And then take care of me when we get there. That I am seen – that is the most important thing to me.* FGD 5.

## Discussion

The aim of this study was to investigate and identify factors and information that older patients find important when planning for their care in order to prepare for patient-centred care. The results show that what matters most to the older patients included in this study relates to respect for autonomy, do no harm, and a holistic approach. These are three comprehensive concepts, which are explained by their categories and subcategories.

Patients’ desire for autonomy is largely related to participation in healthcare, which includes taking part in the planning of care, participation in decision making, and the ability to communicate their own wishes and needs first-hand to all persons actively involved in their care. Participation in one’s care and health care decision making is protected by Swedish law [21], and is part of the human rights set by the United Nations (UN) [36] and one of the core principles of PCC [12, 37]. Still, this study shows that the patients often did not feel that they were taking part in their care. For example, patients felt that decisions were made without them, they were being transported without knowing where they were going, and they were not asked about their needs before being asked to transition from inpatient care to outpatient care. Participation has been shown to result in improved agreement between the patient and the care provider regarding treatment plans, more satisfaction from patients, and improved health outcomes [37]. Additionally, participation leads to increased motivation as well as reduced stress and anxiety regarding one’s care [38]. This relates to the experiences of the patients included in this study, since they felt they sometimes had to suffer unnecessarily because they were unable to communicate their wishes and needs. According to the 1997 Vienna Recommendations on Health-Promoting Hospitals, participation in care is recognized by the WHO as leading to better health and quality of life [39]. Despite this, it has been challenging to implement adequate participation in clinical practice [37], and the patients in our study appear to have still been left on the sidelines of their own care, according to their experiences.

Closely related to “Participation in healthcare” is the wish to be well-informed about one’s care. The importance of this to our patients is in accord with previous studies where patients were asked about requests regarding emergency care [15]. The strive for improved information, communication and patient education is another principle adopted by the WHO through the Vienna Recommendations to improve quality of care [39]. The patients in this study emphasized the importance of correct information to both themselves and their close relatives and/or friends, and the need for transparency in information. This is important for participation as, to be able to participate in healthcare, a patient needs to be well informed about their care and the available alternatives [40]. The need for recurrent and accessible information is even more crucial in situations where many health care professionals are involved in the patient’s care, which is often the case for older patients living with frailty [13]. Having multidisciplinary meetings, as the patients in this study requested, may be a step towards reducing the risk of care fragmentation.

Patients expressed that it mattered to feel trust, safety and security in their care. Factors such as an opportunity for a follow-up visit and continuity were discussed in the FGDs as contributing to these feelings. In today’s strained medical system, follow-up visits and continuity are not always achievable. Apart from reducing the patient’s feelings of safety and security, discontinuity puts the patient at risk for lower perceived wellbeing. Follow-up visits often have the aim to evaluate the patient’s recovery, but they have also been associated with a reduced risk for readmission [41, 42]. The readmission risk seems to be particularly increased during the first few days after discharge [42]. In light of this, a follow-up visit within the first week may not only benefit the patient’s emotional health but also be financially beneficial, as well as reduce unnecessary suffering.

Being given time to adjust to new care plans, decisions or conditions also mattered to the patients in terms of feeling secure about their care. In comparison with the abovementioned factors, this is not necessarily as difficult to achieve. The patients asked to be informed prior to changes, such as transfers between departments or being taken for a CT scan, or prior to an intervention taking place. If health care workers took their time to inform the patients of such changes in advance this would make a big difference in the patients’ experience of their care. Another change that was mentioned was transfer between in- and outpatient care. To be informed beforehand was not only a matter of reducing emotional distress for the patient, but also facilitated a safe and well-coordinated transfer. Procedures for safe and coordinated transfers between in- and outpatient care are specified by law in Sweden [43]. Still, patients experience unnecessary suffering due to rushed discharges and poor planning. Improving this and taking the patients’ needs and wishes into account when planning for a discharge is of utmost importance to provide safe and good quality care.

Accessible care was another factor related to “Trust in the health care services” that mattered to the patients. This has also been reported in previous studies where patients were asked about home-based and emergency care [15, 19]. Article 25 of the UN Universal Declaration of Human Rights declares the right to access to medical care [36]. The WHO states five core concepts regarding the right to health: availability, accessibility, assessment, acceptability, and quality. Together, these constitute the right to accessible, good quality ethical care for all, regardless of geographic location or financial status. This includes the right to sufficient health care facilities and services, such as hospital beds and appointments [44]. The shortage of hospital beds and long waiting times for appointments were discussed throughout the FGDs. As this is a political topic in Sweden, it would be difficult for individual health care professionals to influence these factors. However, factors such as long waiting lists should be considered when planning for the patient’s care. The patients appreciated when someone took the time to explain why aspects of their care could not be carried out within a more reasonable time frame.

Regarding “A holistic approach”, the patients expressed the importance of a good perceived wellbeing. Independence, their living conditions and living in their own homes for as long as possible were all things that mattered to them. Independence is greatly limited by frailty, and frailty is associated with a higher caregiver burden [11]. Putting greater effort into preventing frailty and maintaining independence by making necessary adjustments in daily life would benefit patients’ perceived wellbeing as well as the health care economy. Independence reduces the need for long-term care, which is a major reason for increasing health care provision in the ageing population [4, 6]. Moreover, assessing frailty is an important part of achieving individualized care, but implementing this routine in a clinical setting has proved to be challenging [35].

Attentive care was another factor that mattered to the patients. It was important to them to have helpful carers that listen to and respond to their needs and wishes. The patients wished to be seen and heard, and for health care personnel to consider their emotional health. The WHO states that every person has the right to ethical health care [44]. Ethical health care assures that care is in accordance with the values important to a society or an individual. The WHO further states that the values typically invoked in health care ethics are autonomy, fairness, trust, equity, compassion, honesty, solidarity, freedom and respect [45]. Regrettably, the patients in our FGDs expressed that they did not always experience being treated ethically. Striving to apply ethical care would be both a sensible and an effective way of improving the quality of care for older patients, and for all patients. Regarding the patients’ emotional health, mental health issues are common in older patients, often aggravated by loneliness and social isolation. Most patients receive treatment outside of specialized psychiatric care, but for those who do receive specialized care, the most common diagnosis is depression and addiction in older women and men, respectively [6]. In light of this, it is of great importance to consider emotional aspects when designing a patient-centred individual care plan.

Our results align with previous studies asking older patients living with frailty what mattered most to them relating to urgent care [16, 18]. Autonomy and security were two main categories in a study conducted in the United Kingdom [16], whereas information and safe transitions at discharge where two main themes in another study from England [18].

Despite the research question being broad, most subcategories generated related to codes generated from several different FGDs. The patients seemed to agree on what mattered most, as the same topics arose in different FGs independently from one another. Only a few topics, including “quality of sleep”, “place of death” and “pain management”, were mentioned in only one FGD. All participants were receiving Comprehensive Geriatric Assessment-driven care when the interviews took place, and pain and sleep may have been treated medically, hence not brought up as a concern during the interviews.

To assure validation, confirmability and credibility, the coding and categorization process was discussed between the second and first author (KJ and ÅA) [23]. To improve the quality of the analysis, a researcher more experienced in qualitative methods (KH) was consulted during the process. The study applied broad inclusion criteria to achieve a broad study population. However, the exclusion of the most acutely ill patients and patients who were cognitively unable to participate in FGDs was a clear limitation. As these patients are likely to require more care, their point of view is important.

To increase dependability, a semi-structured interview guide was employed in the FGDs. This was tested in a pilot FGD, after which no changes were made. Previous research has recommended using FGDs to explore patient perspectives [46]. Additionally, using FGDs in pilot studies has been shown to provide a good initial model of the phenomenon studied, which subsequently may be applied in further research using individual interviews for enriching the phenomenon, as well as improving trustworthiness [47]. However, the use of focus groups could also have hindered potential study participants who did not want to share their thoughts with others and reduced the possibility of anonymity. Small focus groups may have limited deep discussions within the group. These considerations also need to be taken into account when choosing a data collection method.

## Conclusions

What mattered most to the patients when planning for their care were that they maintained autonomy, that no harm was done and that the care had a holistic approach. This can also be recognised in WHO’s recommendations of ethical and good quality health care. However, implementation in clinical practice is not yet fully achieved. To acquire a deeper understanding of what matters most to old people living with frailty, individual interviews is a suggest approach for future research.

## Supplementary Information

Supplementary Material 1.

## Data Availability

The datasets generated and analysed in this study are not publicly available. Enquiries for data access should be sent to the corresponding author, at email asa.andersson@oru.se, who will then contact the Ethics Board at Örebro University for permission to openly share the data.

## Abbreviations

CCI: Charlson comorbidity index
CFS: Clinical frailty scale
FGD: Focus group discussion
PCC: Patient-centred care
UN: United Nations
WHO: World health organization
