# Supplementary material for: What matters most to the patient – a qualitative study of older patients in a geriatric ward
Source: BMC Geriatr. 2026 Apr 30;26:611. doi: 10.1186/s12877-026-07555-y (PMC13130787; doi:10.1186/s12877-026-07555-y)
Supplement: Supplementary file 1 — Supplementary Material 1. [file 12877_2026_7555_MOESM1_ESM.docx]

**Interview guide**

What matters most to you when we plan for your care?

Is there anything that is particularly important?

Can you tell us about what is important to you in

- hospital care?
- planned care?
- life in general?

Has the care been adapted to your wishes in consultation with you?

Have you been involved in decisions about your care and treatment?

Are you involved in decisions to the extent you wish?

In what way and in what context?

How would you like it to be?

The questions above refer to:

- during your stay at the department of Geriatrics and after discharge from here.

What matters most to you of the things that have been mentioned here today?
